# Moderated Mediation of Disgust in the Relationship Between Political Ideology and Food Brand Perceptions

**DOI:** 10.21500/20112084.7322

**Published:** 2025-11-19

**Authors:** Alejandro Magallares, Cristian Catena-Fernández

**Affiliations:** 1 Spanish Open University (UNED). School of Psychology, Department of Social Psychology. Madrid, España. Spanish Open University (UNED) School of Psychology Department of Social Psychology Madrid España

**Keywords:** Disgust, experiment, food brand, health risk, political ideology, taste, Asco, experimento, marca alimentaria, riesgo para la salud, ideología política, sabor

## Abstract

The current study investigates whether political appeals of food brands may impact how consumers perceive the healthiness and tastiness of the companies' products. 455 participants (Psychology students, 71% identified as female) read a short and fictitious newspaper story focused on rightists' values (Papa John's Condition), neutral values (Hershey's Condition), or leftists' values (Ben & Jerry's Condition). The confidence interval for the index of moderated mediation showed that the mediation of disgust in the relationship between U.S. food brand and health risk and taste perceptions was moderated by political ideology. Specifically, leftist participants perceived right-wing brands (Papa John's) as less healthy and more disgusting compared to neutral or left-wing brands. No significant effects were observed among rightist participants. This research provides partial support for the idea that "we eat what we are", as leftist participants perceived comfort food products with incongruent political appeals as worse, primarily through disgust mechanisms.

## Resumen

El presente estudio investiga si los mensajes políticos de las marcas alimentarias pueden influir en cómo perciben los consumidores la salubridad y el sabor de los productos de las empresas. 455 participantes (estudiantes de psicología, 71% identificadas como mujeres) leyeron una noticia de periódico breve y ficticia centrada en los valores de los derechistas (condición Papa John's), los valores neutrales (condición Hershey's) o los valores de los izquierdistas (condición Ben & Jerry's). El intervalo de confianza para el índice de mediación moderada demostró que la mediación del asco en la relación entre la marca de alimentos estadounidense y las percepciones de riesgo para la salud y sabor fue moderada por la ideología política. Específicamente, los participantes de ideología de izquierda percibieron las marcas asociadas con la derecha (Papa John's) como menos saludables y más desagradables en comparación con las marcas neutras o de izquierda. No se observaron efectos significativos entre los participantes de derecha. Esta investigación proporciona apoyo parcial a la noción de que "comemos lo que somos", ya que los participantes de izquierda percibieron los productos de comida reconfortante con un posicionamiento político incongruente como peores, principalmente a través de mecanismos de asco.

## 1. Introduction

In 2021, the “We are what we eat” book by the chef Alice Walters suggested the idea that the food we consume influences our moods. Scientific research seems to back this interesting claim as recent fin- dings show that organic food makes us happy and hopeful (Puska et al., 2018), and that there is a relationship between the consumption of spicy food and aggressive cognitions (Batra et al., 2017). But can it happen the other way around? That is, may our attitudes and beliefs shape the evaluation of the food we consume? For example, recent studies have found that conservatives show a preference for naturalness and tradition in food products (Tiganis et al., 2023). In other words, is it possible that “we eat what we are”? That is precisely the main objective of the present research. Specifically, the current study investigates how political appeals of food brands may impact how consumers perceive the healthiness and taste of the companies' products.

### 1.1 Political ideology and food consumption

Recent studies have begun to explore whether alignment with consumers' political values may affect the way people perceive different brands (for a review, see Adaval & Wyer, 2022). Regarding the variables of interest of our study, previous findings show that leftists (e.g., liberals) perceived higher health risks in food products when exposed to claims framed with rightists' (e.g., conservatives) appeals (Boeuf, 2019, Study 1), and that people (e.g., U.S. participants that identified as Southerners) expected that identity-congruent foods (e.g., grits) would be tastier prior to consumption (Hackel et al., 2018, Study 1).

(Boeuf 2019, Study 2) found that the mechanism that would explain why leftists perceived less risk in left-wing products was the fluency (e.g., it is easier to understand messages that are congruent with our previous attitudes), but recent studies suggest that the variable that may be mediating the relationship between political ideology and food perception is the disgust elicited by these products (Tal et al., 2022, Study 2).

### 1.2 Political ideology and disgust

Recent studies have begun to explore the relationship between disgust and political ideology (for a review, see Petersen et al., 2020). Regarding the food domain, recent investigations suggest that moral transgressions that clashed with political ideologies held by consumers (e.g., a cup-cake that may be considered “racist”, as its shape resemble a Ku Klux Klan member) produced disgust, which in turn undermined the expected taste of a product (Tal et al., 2022, Study 1).

It is important to remark that the connection between health risk perception, taste, and disgust makes sense from an evolutionary point of view (for a review, see Tybur et al., 2013). For example, some elements of taste (e.g., bitterness) may have evolved to detect potential health risks (e.g., pathogens or poison) of certain foods (e.g., rotten foods or poisonous plants). These foods elicit an unpleasant taste sensation (e.g., bitter), which triggers a set of physiological (e.g., nausea) and behavioral responses (e.g., opening of the mouth) that prevent the ingestion of the perilous food that may be a health risk that can endanger our life (Curtis & Biran, 2001).

### 1.3 Research overview

Both the investigations by Boeuf (2019) and Tal et al. (2022) measured health risk perception and taste but with “fake” products (invented hot dogs and fictional cupcakes, respectively). In our research we went a step further by analyzing whether we would find similar results with real products.

The ideological asymmetry between leftists and rightists extends to consumer choices (DellaPosta et al., 2015). This political divide has been observed to polarize consumers between liberals vs. conservatives brands (Schoenmueller et al., 2023). Specifically, rightists and leftists have different food preferences, and polls show us, for example, that while the former usually favor Papa John's, the latter prefer Ben & Jerry's (Axios Harris Poll, 2019, 2022). Meanwhile, other companies, such as Hershey's, are usually perceived as a trustworthy brand by both leftists and rightists (Axios Harris Poll, 2019, 2022).

These three companies represent the preferred comfort food products consumed in the U.S. For example, 15% of U.S. citizens list pizza (e.g., Papa John's) as their favorite comfort food, while chocolate (e.g., Hershey's) and ice cream (e.g., Ben & Jerry's) tied for second, each garnering 7% (Harris Poll, 2016).

We believe, according to the reviewed literature, that associating a comfort food product with an “objectionable” ideology (e.g., incongruent with our previous political attitudes) should undermine their healthiness (Boeuf, 2019) and tastiness (Tal et al., 2022). Furthermore, similar to the rejection of “suspicious” foods caused by physical disgust (Curtis & Biran, 2001), we think that “ideological” disgust (e.g., the negative emotion we feel when we are ex- posed to an incongruent political message) may lead to a worse perception of the pro- duct (more health risk perception and less taste) (Tal et al., 2022). Finally, we decided to test a moderated mediation model, as other authors have recently done (Boeuf, 2019; Hackel et al., 2018), to see whether the effects of the comfort food brand on our variables of interest (health risk perception and taste) were mediated by disgust, con- tingent on political ideology.

## 2. Method

### 2.1 Participants

We recruited 519 psychology students (for this research, only students enrolled in this specific degree program were selected) from the Spanish Open University (UNED) who volunteered to participate in an on-line study via Qualtrics. Participants who completed the questionnaire in less than two minutes or who responded incorrectly to the attention check (“Plea- se select “strongly agree” to show you are paying attention to this question”) were excluded from the analysis. The final sample comprised 455 participants (326 leftists, 129 rightists). We did not determi ne the sample size a priori. A sensitivity analysis conducted with G*Power (Faul et al., 2009) specifying six groups (see more information in the Procedure Section) and two degrees of freedom (ANOVA: Fixed effects, special, main effects, and interactions; a = .05) revealed that this sample provided 80% power to detect effects greater than Cohen's f = .146.

### 2.2 Procedure

Participants (71% identified as female) received an email in which they were invited to participate in a survey about how people perceived a U.S. food brand. Once they gave their informed consent, we first asked them to answer sociodemographic variables (age and gender). Participants (Mage = 39.25, SDage = 11.24) were then randomly allocated to one of the three conditions regarding the U.S. food brand, namely: Papa John's versus Hershey's ver sus Ben & Jerry's.

Participants read a short and fictitious newspaper story (based on previous statements of the real founders of the companies, see Appendix) focused on rightists' values (Papa John's Condition), neutral values (Hershey's Condition), or leftists' values (Ben & Jerry's Condition). The manipulation text in the three conditions was as follows:

*“Papa John's [vs. Hershey's vs. Ben & Jerry's] founder claims that his conservatives' values [vs. values based on the golden rule vs. progressives' values] made a better pizza [vs. chocolates vs. ice cream]. John Schnatter [vs. Milton Hershey vs. Ben Cohen and Jerry Greenfield] said that “truth in God” [vs. the principle of “treating others as you want to be treated” vs. “social justice”] was a key factor that helped his company emerge as one of the world's largest pizzas [vs. chocolates vs. ice creams] chains”.*

After reading this information, participants responded to the dependent and con trol variables described in the Measures Subsection. Upon completion of the survey, participants were debriefed online about the purposes of the study.

### 2.3 Instruments

If not indicated otherwise, all the items were assessed on 7-point Likert-type scale ranging from 0 = strongly disagree to 6 = strongly agree.

Health risk perception. We asked participants (a = .93 on our sample) to indicate to what extent they thought that the U.S. food brand caused an important risk of: (1) obesity, (2) diabetes, and (3) cardiac illnesses (Cadario, 2016).

Taste perception. To measure the extent to which participants thought the U.S. food brand would be tasty (a = .71 on our sample), we used the following two items (Tal et al., 2022): (1) “I think the [U.S. food brand] has a lot of taste”, and (2) “I would enjoy eating the [U.S. food brand]”.

Disgust perception. We measured the degree to which participants felt dis- gust toward the U.S. food brand with the following item (Tal et al., 2022): “I would feel disgusted by eating the [U.S. food brand]”.

Control variables. We measured previous consumption of the product (on a 7-point Likert-type scale ranging from 0 = never to 6 = always) and we also assessed the brand knowledge (on a 7-point Likert-type scale ranging from 0 = not at all to 6 = a lot) as this study was conducted outside the U.S. .

Manipulation check. We used the following items: (1) “I think that the [U.S. food brand] is a product from a brand that promotes conservative or right-wing values”, (2) “I think that the [U.S. food brand] is a product from a brand that promotes left-wing or progressive values”, and (3) “I think the [U.S. food brand] is a product from a brand that promotes neutral values ( it does not promote progressive or conservative values)”. To check the effective-ness of our manipulation, we ran a one-way ANOVA with the condition (U.S. Food Brand) as the independent varia ble and with each of the three items as the dependent variables.

Results on the first item yielded a significant main effect for U.S. Food Brand (F(2, 452) = 41.35, p < .001, n2 = .155). The Tukey post-hoc test revealed that participants indicated that Papa John's (M = 3.51; SD = 1.48) promoted significantly more conservative or right-wing values than Ben & Jerry's (M = 2.14; SD = 1.22, p < .001) and Hershey's (M = 2.73; SD = 1.24, p < .001).

Results on the second item yielded a significant main effect for U.S. Food Brand (F(2, 452) = 27.73, p < .001, n2 = .109). The Tukey post-hoc test revealed that participants indicated that Ben & Jerry's (M = 2.76; SD = 1.42) promoted significantly more progressive or left-wing values than Papa John's (M = 1.71; SD = 1.21, p < .001) and Hershey's (M = 2.43; SD = 1.14, p = .053).

Results on the third item yielded a significant main effect for U.S. Food Brand (F(2, 452) = 13.12, p < .001, n2 = .055). The Tukey post-hoc test revealed that participants indicated that Hershey's (M = 3.39; SD = 1.33) promoted significantly more neutral values than Papa John's (M = 2.52; SD = 1.80, p < .001), but the differences with Ben & Jerry's were not significant (M = 3.24; SD = 1.58, p = .66). Taken together, these findings confirmed the effectiveness of the manipulation.

Political ideology. We asked participants to identify themselves along a continuum of political ideology ranging from 1 = very left-winger to 10 = very right-winger. We decided to use a 10-point scale (for a discussion, see Kroh, 2007), since having no midpoint, it allowed us to categorize all participants into one of the two sides of the ideological spectrum (Catena-Fernández, & Fernández, 2025a, 2025b): participants who selected 1 to 5 were categorized as leftists (-1) and those who selected 6 to 10 were categorized as rightists (1). This variable was used as an independent criterion variable in the analyses.

## 3. Results

We first present the results of the U.S. Food Brand X Political Ideology ANCOVAs on all dependent variables. Then, we present the results of the moderated mediation models conducted to test the hypothesized conditional indirect effects. In all the analyses, we included the previous consumption of the product and brand knowledge as covariate variables.

### 3.1 ANCOVAs

**Figure 1:**
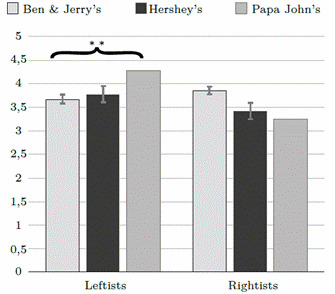
U.S. Food Brand x Political Ideology interaction on health risk perception

The results on health risk perception (see Table 1 and Figure 1) yielded the expected significant interaction (F(2, 453) = 5.49, p < .01, np2 = .024), a non-significant main effect for U.S. Food Brand (p = .50), and a significant main effect for Political Ideology (F(1, 453) = 7.52, p < .01, np2 = .017). The interaction was marked by a significant effect of the U.S. Food Brand among leftists (F(1, 324) = 6.45, p < .01, n2 = .039), but not among rightists (F(1, 128) = 1.45, p = .24, n2 = .023). The Bonferroni post-hoc test revealed that leftists reported significantly more health risk perception in the Papa John's (M = 4.25, SD = 1.00) than in the Ben & Jerry's (M = 3.65, SD = 1.36, p < .01) and the Hershey's conditions (M = 3.75, SD = 1.45, p < .01).

**Table 1:** Results of the moderate mediation analyses

Conditional indirect effects on health risk perception				
Political Ideology	Effect	Boot SE	Boot LLCI	Boot ULCI
Leftists	.092	.032	.040	.163
Rightists	-.032	.052	-.145	.063
Index of moderated mediation				
Moderator Index		Boot SE	Boot LLCI	Boot ULCI
Political Ideology	-.026	.064	-.265	.015
Conditional indirect effects on taste				
Political Ideology	Effect	Boot SE	Boot LLCI	Boot ULCI
Leftists	-.165	.048	-.267	-.075
Rightists	.056	.089	-.112	.233
Index of moderated mediation				
Moderator Index		Boot SE	Boot LLCI	Boot ULCI
Political Ideology	.222	.103	.026	.413

*Note*. Bootstrap samples = 5,000; LLCI = lower level of the 95% confidence interval, ULCI = upper level of the 95% confidence interval.

Contrary to our expectations, the results for taste perception yielded a non-significant interaction (p = .18), a significant main effect for U.S. Food Brand (F(2, 452) = 6.13, p < .05, np2 = .027), and a non-significant main effect for Political Ideology (p = .59).

**Figure 2:**
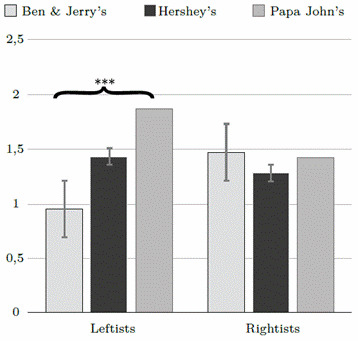
U.S. Food Brand x Political Ideology interaction on disgust

*Note*. mp < .001

The results on disgust perception (see Table 1 and Figure 2) yielded the expected significant interaction (F(2, 453) = 2.95, p = .05, n^p^2 = .013), a non-significant main effect for U.S. Food Brand (p = .06), and a non-significant main effect for Political Ideology (p = .99). The interaction was marked by a significant effect of the U.S. Food Brand among leftists (F(1, 324) = 8.08, p < .001, n2 = .048), but not among rightists (F(1, 128) = 1.12, p = .33, n2 = .018). The Bonferroni post-hoc test revealed that leftists reported significantly more disgust in the Papa John's (M = 1.87, SD = 1.50) than in the Ben & Jerry's (M = 0.95, SD = 1.28, p < .01) and the Hershey's conditions (M = 1.43, SD = 1.28, p < .05).

#### Moderated mediation models

We used Hayes' (2017; model 7, 5,000 boots-trap resampling) PROCESS macro for IBM SPSS (Version 27, 2020) to test whether the effects of the U.S. food brand, (1 = Papa Jo- hn's, 0 = Hershey's, -1 = Ben & Jerry's) on health risk perception/taste were mediated by disgust, contingent on political ideology (1 = rightists, -1 = leftists). We therefore defined two moderated mediation models (one for health risk perception and one for taste as a dependent variable), considering ideology as the moderator of the indirect effects of U.S. Food Brand on the respective dependent va riable via disgust. Previous consumption of the product and brand knowledge were entered as covariates for each model.

**Figure 3:**
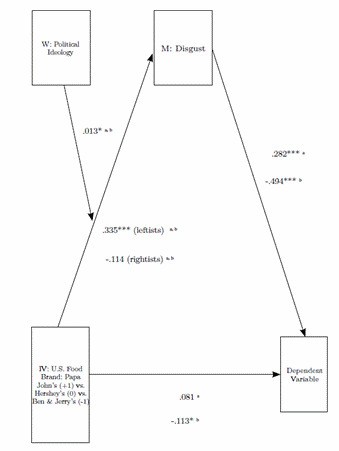
The multiple moderated mediation analysis (model 7 by Hayes, 2017)

*Note*. A Model with health risk perception as a depen- dent variable; bModel with taste as a dependent va riable. Interactions, *p < .05; **p < .01; ***p < .001

As expected, the confidence interval for the index of moderated mediation confirmed that the mediation of disgust in the relation between U.S. food brand and health risk perception/taste was moderated by political ideology (see Table 2 and Figure 3). In the model in which the dependent variable was health risk perception, conditional indirect effects indicated that disgust acted as mediator among leftists (b = .094, SE = .032, 95% CI: [.040, .163]) but not among rightists (b = -.032, SE = .052, 95% CI: [-.145, .063]). In the model in which the dependent variable was taste, conditional indirect effects indicated that disgust acted as mediator among leftists (b = -.165, SE = .048, 95% CI: [-.267, -.075]) but not among rightists (b = .056, SE = .089, 95% CI: [-.112, .233]).

**Table 2:** Means and standard deviations of all the dependent variables across conditions

Mean (SD)						
Dependent Variable		Leftistists			Rightists	
	Ben & Jerry's	Hershey's	Papa John's	Ben & Jerry's	Hershey's	Papa John's
Health risk	3.65	3.75	4.25	3.82	3.40	3.23
perception	(1.36)	(1.45)	(1.00)	(1.48)	(1.64)	(1.57)
Taste	4.38	3.84	3.49	4.10	4.04	3.83
	(1.25)	(1.06)	(1.22)	(1.44)	(1.05)	(1.26)
Disgust	.95	1.43	1.87	1.47	1.28	1.42
	(1.28)	(1.28)	(1.50)	(1.74)	(1.34)	(1.68)

*Note*. All the dependent variables scores range from 0 = strongly disagree to 6 = strongly agree.

## 4. Discussion

Our results showed that leftists reported significantly more health risk perception in the Papa John's condition. Furthermore, leftists reported significantly more disgust perception in the Papa John's condition. However, contrary to our expectations, the results for taste yielded a non-significant interaction. Our findings corroborate prior research demonstrating the pervasive influence of political ideology-specifically, leftist ideology in this study- on societal domains, extending its impact to consumer behavior within the food marketplace.

In addition, disgust acted as a mediator between U.S. food brand and health risk perception among leftists, but not among rightists. A similar pattern was found in the case of taste. These results suggest the role of disgust as the potential underlying mechanism for brand rejection (Tal et al., 2022). We believe that this study suggests that food companies may wish to exercise caution when creating political associations for their products.

In spite of the findings obtained, there are some limitations to the present research that should be considered. Firstly, a critical limitation is the substantial imbalance in our sample (326 leftists vs 129 rightists, 71% female), which may have limited our statistical power to detect effects among rightists and raises questions about the generalizability of our findings. The predominance of leftist participants means that our results primarily reflect leftist responses to politically incongruent brands, rather than a symmetrical effect of political ideology. A further limitation is the exclusive use of on-line data collection procedures. While some researchers have raised concerns regarding the validity of web-based studies, adherence to expert recommendations, as implemented in this research, can effectively mitigate potential issues (for a review, see Reips 2021).

## 5. Conclusión

This research provides evidence that leftist consumers may avoid eating what contradicts their political identity, as leftist participants perceived comfort food products with right-wing appeals as worse (higher health risk perception, more dis- gust, lower expected taste). However, the absence of reciprocal effects among rightist participants suggests caution in generalizing this pattern. Given this is a single study, future research should continue to explore this line of inquiry to determine if similar results can be replicated.

## 6. Ethics statement

The research was conducted in accordance with the ethical principles stated in the Declaration of Helsinki. The observational and non-invasive nature of the online study did not require formal approval by UNED's institutional review board.

Participants in the final sample consented to participate in the study, and they were allowed to withdraw from the study whenever they wanted. The data were collected anonymously, and results were reported in aggregate form only, so that participants could not be identified individually.

## 7. Declaration of Competing Interest

The authors declare that they have no known competing financial interests or personal relationships that could have appeared to influence the work reported in this paper.

## 8. Data availability

Data will be made available on request.

## 9. Funding

No funding was received.

## 10. Authorship contribution statement

AM contributed to the study conception and design. Material preparation and data collection were performed by CCF. Analyses were performed by CCF. The first draft of the manuscript was written by AM. All authors read and approved the final manuscript.

## Appendix

John Schnatter (the original founder of Papa John's) has declared that their pizzas have gotten worse without his “conservative values” (after some racists remarks, he was removed from the company in 2018). In addition, Schnatter has also suggested that “truth and God” made Papa John's great (Insider, 2022).

When Milton Hershey (the original founder of Hershey's) was asked once what his religion was, he is said to have replied, “the golden rule” (Hershey's webpage, 2023). The golden rule (“Do unto others as you would have them do unto you”) may be perceived as “conservative” or a “tradition” (e.g., messages about the golden rule are common in many religions) but at the same time as a “progressive value” related to “benevolence” (e.g., helping people in need) (Schwartz et al., 2014).

On the Ben & Jerry's website (2023), the company declares their own “progressive” values, claiming that they support causes like “racial justice”, “rights and dignity of refugees”, or “democracy” (among others). The founders of the company (Ben Cohen and Jerry Greenfield) have been very vocal supporting different “social justice” campaigns in the media (e.g., Wall Street Journal, 2022).
